# Structural Analysis of HIV-1 Maturation Using Cryo-Electron Tomography

**DOI:** 10.1371/journal.ppat.1001215

**Published:** 2010-11-24

**Authors:** Alex de Marco, Barbara Müller, Bärbel Glass, James D. Riches, Hans-Georg Kräusslich, John A. G. Briggs

**Affiliations:** 1 Structural and Computational Biology Unit, European Molecular Biology Laboratory, Heidelberg, Germany; 2 Department of Infectious Diseases, Virology, Universitätsklinikum Heidelberg, Heidelberg, Germany; Northwestern University, United States of America

## Abstract

HIV-1 buds form infected cells in an immature, non-infectious form. Maturation into an infectious virion requires proteolytic cleavage of the Gag polyprotein at five positions, leading to a dramatic change in virus morphology. Immature virions contain an incomplete spherical shell where Gag is arranged with the N-terminal MA domain adjacent to the membrane, the CA domain adopting a hexameric lattice below the membrane, and beneath this, the NC domain and viral RNA forming a disordered layer. After maturation, NC and RNA are condensed within the particle surrounded by a conical CA core. Little is known about the sequence of structural changes that take place during maturation, however. Here we have used cryo-electron tomography and subtomogram averaging to resolve the structure of the Gag lattice in a panel of viruses containing point mutations abolishing cleavage at individual or multiple Gag cleavage sites. These studies describe the structural intermediates correlating with the ordered processing events that occur during the HIV-1 maturation process. After the first cleavage between SP1 and NC, the condensed NC-RNA may retain a link to the remaining Gag lattice. Initiation of disassembly of the immature Gag lattice requires cleavage to occur on both sides of CA-SP1, while assembly of the mature core also requires cleavage of SP1 from CA.

## Author Summary

HIV-1 buds from the plasma membrane of infected cells in an immature form with the polyprotein Gag as its major component. Maturation into an infectious form requires cleavage of Gag in five positions. This process is an important target for antiretroviral drugs. Here we studied changes in the structure of the virus that occur during maturation, making use of virus variants in which different combinations of cleavage sites were mutated to prevent cleavage at those sites. We used cryo-electron tomography and sub-tomogram averaging to visualise the arrangement of Gag in 3D. We show that the fastest cleavage event leads to condensation of the RNA genome complexed with viral proteins. This inner RNA/protein structure appears to maintain a link with the remaining Gag lattice. Processing on both sides of CA-SP1, the main structural module of Gag, is required for disassembly of the immature Gag lattice, while removal of SP1 is needed in addition for mature core formation. The results provide structural correlates of the ordered processing events during HIV-1 maturation and shed light on the mechanism of action of bevirimat, an inhibitor of CA-SP1 cleavage in clinical trials.

## Introduction

**Figure 1:**
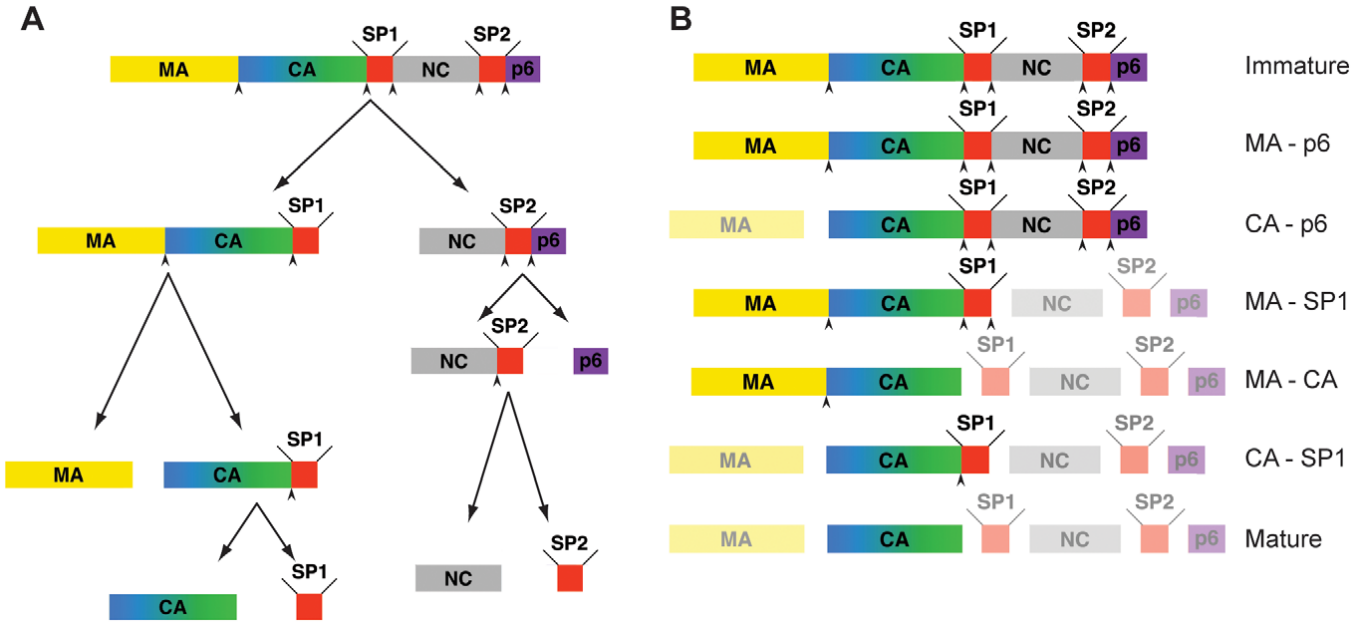
Steps in HIV-1 proteolytic maturation, and variants analysed. A) Schematic outline of the proteolytic cleavages which take place in Gag during the HIV-1 maturation process. Arrowheads indicate proteolytic sites before cleavage. The order of cleavage events shown is based on the rates of cleavage in vitro as described in [22]. B) Schematic representation of Gag after completion of cleavage for each variant analyzed. The non-cleaved products due to the inactivation of the proteolytic sites are highlighted. Mutated, and therefore uncleaved processing sites are indicated by an arrowhead.

The assembly of HIV-1 occurs at the plasma membrane of infected cells. The primary structural component of the assembling virus is the 55-kDa polyprotein Gag. Multiple copies of Gag assemble to form an incomplete sphere underneath the plasma membrane, which recruits components of the cellular ESCRT machinery to mediate membrane scission and release of the budding virus from the cell [1], [2], [3]. Gag consists of three major structural components: MA, the membrane binding domain, CA, the capsid domain, and NC, the nucleocapsid domain, which interacts with the viral RNA. CA and NC are separated by a short linker peptide SP1, and downstream of NC are two further peptide domains, SP2 and p6 (Figure 1A) [4]. p6 contains the short linear motifs which are responsible for ESCRT recruitment [2], [5], [6]. The viral genome is recruited to the assembling particle from the pool of cellular mRNA via interactions between a packaging signal (Ψ) present at the 5′ end of the genome [7] and zinc-fingers present in NC [4].

During or concomitant with budding, the viral protease (PR) cleaves Gag in five positions into its component domains. These cleavages lead to the dramatic morphological changes required for creation of a mature, infectious virion. Proteolytic maturation is essential for infectivity, and PR inhibitors are a key element of current antiretroviral therapies [8]. Maturation appears to be a rapid process, and viruses imaged by electron microscopy (EM) in the vicinity of infected cells are predominantly mature, with only occasional immature particles. Intermediate maturation states have not been detected so far. For this reason, the majority of morphological and structural studies have focused either on the mature virus, or on the immature virus that can be produced in the presence of a PR inhibitor or by mutation of the PR active site.

In the immature virus, Gag is arranged in a radial fashion, with the N-terminal MA domain associated with the viral membrane, and the C-terminus of the protein pointed towards the centre of the particle. Gag adopts a curved hexameric lattice with an 8 nm unit cell [9], which closes through the incorporation of heterogeneously shaped defects [10]. After proteolytic cleavage, the MA layer is thought to remain associated with the viral membrane, whereas NC and the RNA are condensed into a ribonucleoprotein complex layer surrounded by the viral CA capsid core. The core is most often cone-shaped, though other shapes including capped tubes are seen. The core is thought to have a fullerene cone geometry formed from a hexameric lattice with twelve pentamers incorporated to allow closure [11], five at the narrow end of the cone and seven at the broad end of the cone shaped structure. CA adopts a larger 10 nm unit cell in the mature core [12], [13].

The structure of the immature lattice has been explored by cryo-electron tomography combined with sub-tomogram averaging [10], [14]. The N-terminal CA domain forms hexameric rings surrounding central holes. Below these rings, 2-fold symmetric densities link adjacent hexamers. These densities have been assigned to the dimeric C-terminal CA domain. Six such dimers converge below the holes in the N-terminal CA domain into a rod-like structure suggested to represent a six-helix bundle formed by SP1 [14]. This rod-like structure descends towards the centre of the virus into the NC-RNA layer, which is not hexagonally ordered [10].

Studies of the arrangement of CA in the mature core are much more advanced through the use of in vitro assembly of purified proteins to form regular arrays appropriate for high-resolution structural study [15], [16]. In the mature core, the N-terminal residues of CA are folded back into the structure to form a β-hairpin [17]. The formation of this structure appears to be conserved in all retroviruses with the exception of the spumaviruses [17], [18], [19], [20]. Mutagenesis studies suggest that the formation of the β-hairpin is important for the formation of the mature core [17], [21]. This structure cannot be present in the immature virus since the N-terminal residues are covalently linked to the MA domain.

The five proteolytic cleavage sites in Gag are cleaved at very different rates in vitro. The fastest cleavage is that between SP1 and NC. Relative to this cleavage, SP2-p6 is cleaved 9x slower, MA-CA 14x slower, NC-SP2 350x slower, and CA-SP1 400x slower [22]. These differences, together with the observation of distinct and reproducible processing intermediates in lysates from infected cells or upon partial inhibition of PR [23], suggest that cleavage is a stepwise process in vivo. The order of cleavage based on these rates determined in vitro is illustrated in Figure 1A with initial processing separating NC-p6 from the membrane-bound N-terminal part of Gag, secondary cleavages separating MA from CA-SP1 and p6 from NC-SP2 and final processing removing the two spacer peptides from the C-termini of CA and NC, respectively. Mutational studies revealed that the efficacy and order of these cleavage events is highly relevant for viral infectivity: blocking cleavage at any individual processing site - with the exception of NC-SP2 - severely diminished or abolished virion infectivity [24], [25], [26], [27], mostly affecting fusion or early post-fusion events [24], [25], [28].

Due to the fast time scale of maturation and the absence of any system for synchronizing the process, studies of maturation intermediates have relied upon viruses in which individual cleavage sites have been mutated [22], [27], [29], [30]. These studies showed that mutation of the cleavage site between MA and CA led to normal condensation of the NC-RNA complex without formation of the cone shaped capsid [27], [29]. Inhibition of both cleavages flanking SP1 led to viruses with immature-like morphology, whereas inhibition of the CA-SP1 cleavage allowed NC-RNA condensation, but prevented formation of a mature-like core [27]. A thin electron-dense layer separated from the virion membrane was observed in this case and suggested to correspond to CA. A similar phenotype was observed when virus was produced in the presence of bevirimat [31], which specifically inhibits cleavage between CA and SP1. Although the inhibition of CA-SP1 processing by bevirimat is incomplete [31], [32], the infectivity of virions raised in its presence is severely diminished. Similarly, partial inhibition of polyprotein processing by co-expression and co-assembly of wild-type (wt) and cleavage-site mutated Gag polyprotein showed a strong trans-dominant effect of the mutated proteins, which appeared strongest for mutations affecting the CA-NC border [25], [26], [33]. Mutations spanning the CA-SP1 regions also had a trans-dominant negative effect on processing of the respective wt protein when co-assembled into particles, suggesting that the CA-SP1 region forms a stable multimeric structure [26].

Here we set out to elucidate and describe the stepwise transitions occurring during HIV-1 maturation on a structural level. By doing so we also aimed to shed light on the structure and integrity of the Gag lattice by understanding structural changes associated with specific cleavage events. To do this we have applied cryo-electron tomography and sub-tomogram averaging to describe the morphology and structure of an extended pool of cleavage site mutants.

## Results

### Virus production and cleavage efficiency

We analyzed a panel of viruses derived from HIV-1 NL43, which carry point mutations at single or multiple of the cleavage sites in Gag [24], [27], [28] to prevent proteolytic cleavage at the targeted sites. In parallel we transfected a wt proviral plasmid in the presence or absence of the PR inhibitor Lopinavir to generate immature and mature viruses, respectively. The constructs used are shown in Figure 1B. Based on the scheme shown in Figure 1A [22], MA-p6 (together with PR inhibited virus), MA-SP1, CA-SP1 (CA5 in [27]) and the wt virus represent true maturation products. The other constructs CA-p6, and MA-CA were selected to shed light on the importance of individual cleavage sites upstream and downstream of CA.

**Figure 2:**
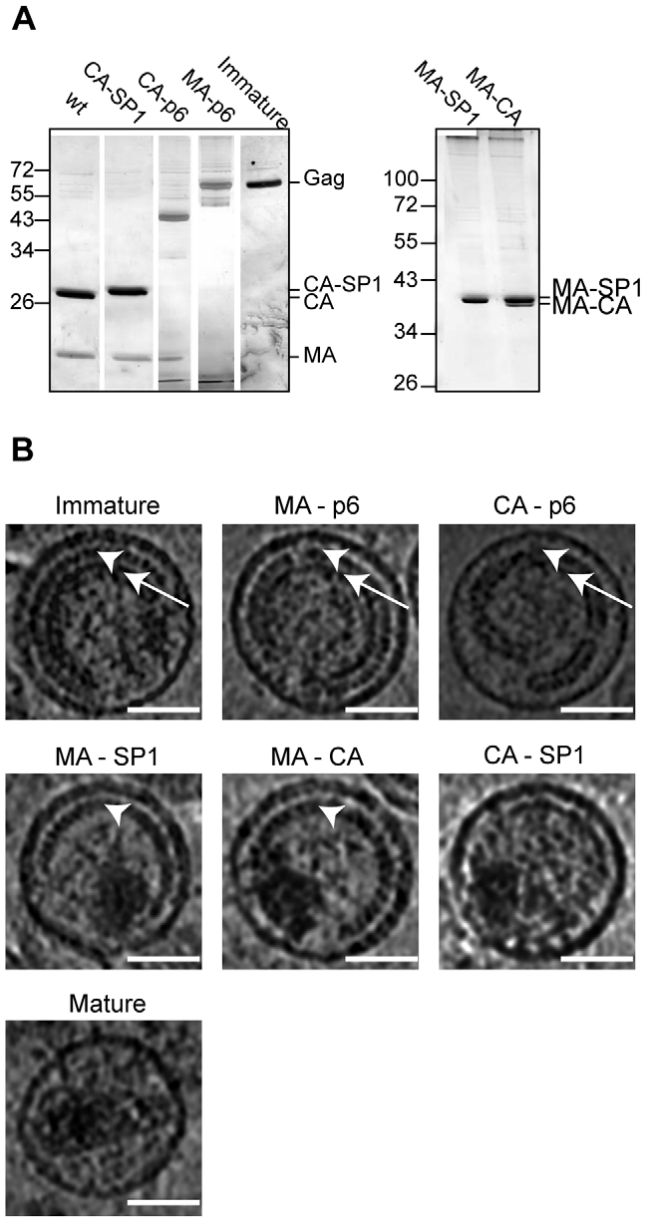
Characterisation of HIV-1 variants. A) Partially processed Gag-derived products detected by SDS-PAGE. Iodixanol gradient purified particle preparations of the indicated HIV-1 variants were separated on 12.5% SDS gels. Virion-associated proteins were visualized by silver staining according to standard procedures, showing MA and CA containing Gag derivatives as the most prominent bands. Lanes in the left panel were combined from two independent gels and were size adjusted for comparison. The right panel illustrates incomplete processing at the CA-SP1 boundary for variant MA-CA. Positions of molecular mass standards (in kDa) are indicated to the left. B) Central sections of 0.8 nm thickness from 6 µm defocus tomographic reconstructions of the HIV-1 variants analyzed. White arrowheads point to the immature CA layer, whereas white arrows point to the NC-RNA layer. Immature, MA-p6 and CA-p6 variants show both CA and NC-RNA layers. In CA-p6 the distance between the CA layer and the membrane is more variable. In MA-SP1 and MA-CA, the CA layer is seen, but the NC-RNA has condensed. CA-SP1 and the mature virus do not show the characteristic striated CA layer. The scale bar is 50 nm.

HIV-1 wt and all variant viruses were produced by transfection of 293T cells, and purified by gradient centrifugation as described previously [10]. We assessed the degree of proteolytic processing via SDS PAGE followed by silver staining or immunoblotting (Figure 2A and data not shown). In most cases all predicted cleavage products were seen, indicating that proteolytic cleavage at non-mutated sites proceeded to completion, while mutated sites were blocked. The exception was MA-CA carrying a single mutation at the cleavage site between MA and CA, but exhibiting strongly reduced cleavage efficiency at the CA-SP1 boundary of only 20%–50% depending on the virus preparation. Failure to cleave at MA-CA therefore leads to a reduction in the efficiency of cleavage at the CA-SP1 boundary.

### 3D morphology of the viruses

In order to characterize the effect of cleavage site mutations on structure and morphology of the viruses, purified viruses were plunge-frozen in liquid ethane and imaged in the electron microscope. To eliminate morphological variability resulting from transfection or purification, a minimum of 3 independent preparations of each virus were imaged. With the exception of the CA-SP1 construct (see below), independent preparations of the same variant showed no significant differences in morphology. In order to obtain three-dimensional structural information, cryo-electron tomograms were acquired. A minimum of 46 virus particles were reconstructed in 3D for each variant, in each case distributed across all 3 preparations.

Figure 2B shows central sections through representative tomograms of viruses from each of the variants (see also Figure S1). Both the PR inactivated wt virus and the MA-p6 variant show the characteristic morphology, which has previously been described for immature HIV imaged by cryo-electron microscopy and cryo-electron tomography [10], [14], [34]. These virus particles are approximately spherical and display an incomplete protein lattice underneath the viral membrane. The membrane appears thicker in regions where Gag is present, due to the presence of the membrane associated MA domain in these regions [10]. The protein lattice shows a striated pattern in the outer ring of density, previously assigned to the CA domain (Figure 2B, arrowheads), and a smoother inner ring of density, previously assigned to the NC-RNA complex (Figure 2B, arrows) [34]. The similarity of the PR-inhibited virus and the MA-p6 variant indicates that there are no substantial morphological differences between a virus which has an active PR, but mutations in all proteolytic cleavage sites in Gag, and a virus with intact cleavage sites but an inhibited PR.

The characteristic two layers of density corresponding to the CA and NC-RNA domains were also seen in the CA-p6 virus; however, the distance between these layers and the membrane was significantly more variable than in the immature virus (compare CA-p6 with immature in Figure 2B), leading to greater variability in the curvature of the layer.

MA-SP1 and MA-CA showed a thinner Gag layer. The striated CA layer of density was present, but the inner NC-RNA layer of density that is seen in the immature virus was missing. Cleavage between CA and NC therefore leads to loss of the NC-RNA layer. Instead we observed a globular dense structure within the virus that is likely to represent condensed NC-RNA. The volume of the condensed NC-RNA was measured by visual assessment of the boundaries of the condensed region. In MA-SP1 it was 4.4±1.1 * 10^4^ nm^3^ (n = 46), and in MA-CA was 4.3±1.2 * 10^4^ nm^3^ (n = 46), corresponding to a ratio between the volume of the virus particle and the volume of the condensed NC-RNA of 26.9±8.5 for MA-SP1 and 28.0±8.2 for MA-CA and thus indicating no detectable difference in the degree of condensation.

The CA-SP1 viruses presented high variability in the morphology depending on the preparation. Of the 5 independent preparations, 2 showed no internal structures, whereas the other 3 showed features, and were further analysed. In contrast to what was observed for the other mutated variants, the striated CA layer was not present in any of the particles, while a discontinuous and thin layer was visible in ∼45% of the viruses (29 of 64 particles). The NC-RNA was condensed to the same degree as in MA-SP1 and MA-CA (volume 4.3±1.0 * 10^4^ nm^3^ (n = 46), ratio of virus volume to NC-RNA volume 26.4±7.2). The membrane appears thinner, similar to the regions of the immature virus where Gag is absent.

The mature virus showed the same morphology as previously described by tomography of equivalent preparations [35]. Virus particles contained predominantly cone shaped cores consisting of a thin CA lattice containing density which we attribute to the NC-RNA. The NC-RNA volume was 3.5±0.8 * 10^4^ nm^3^ (n = 46), corresponding to a ratio between the volume of the virus particle and the volume of the condensed NC-RNA of 35.0±8.9. The slightly lower NC-RNA volume in the mature virus when compared to MA-SP1, MA-CA and CA-SP1 probably reflects the better definition of the NC-RNA edges when confined within the core, rather than a change in the degree of condensation. The membrane thickness appears similar to that in the CA-SP1 viruses.

### Global arrangement of the Gag lattice

**Figure 3:**
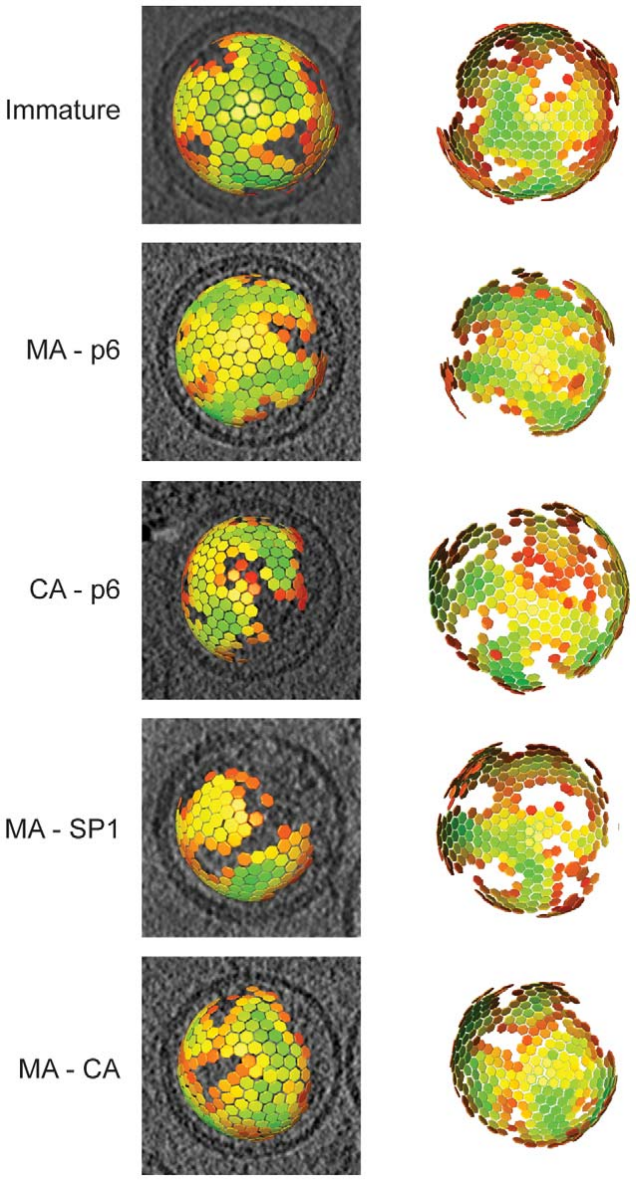
The global arrangement of the Gag layer. Global lattice maps for each variant superimposed on central sections of the tomographic reconstruction of the virus (left panels), or viewed from the direction of the largest gap in the lattice (right panels). The centres of each hexameric unit cell are marked with hexamers, which are coloured according to cross correlation on a scale from low (red) to high (green). Higher cross correlation values indicate that the subtomogram is more similar to the average structure. The cross-correlation range in each map has been set between the minimum and the maximum cross-correlation value present in the map. Maps are shown in perspective such that hexamers on the rear surface of the particle appear smaller.

To describe the changes in the global arrangement of the Gag lattices in greater detail, we applied subtomogram averaging to the cryo-electron tomography data for those variants which showed a striated lattice (the thinner CA layer in CA-SP1 and wt proved insufficiently featured for subtomogram averaging). By plotting in 3D the position of the subtomograms after the alignments, we were able to define the position and the orientation of the centre of each identified hexagon of the lattice. All the lattice maps showed the same general arrangement of a continuous hexameric lattice with irregularly shaped defects, partially covering the inner surface of the viral membrane, but leaving one large gap (Figure 3). There was no substantial difference in the degree of shell completeness between the different variants.

### Structure of the Gag lattice by sub-tomogram averaging

To describe the changes in the Gag lattice structures during the maturation process in greater detail, we analysed the average structures generated during subtomogram averaging. These represent a 3D reconstruction of the local structure of the Gag lattice in each variant.

**Figure 4:**
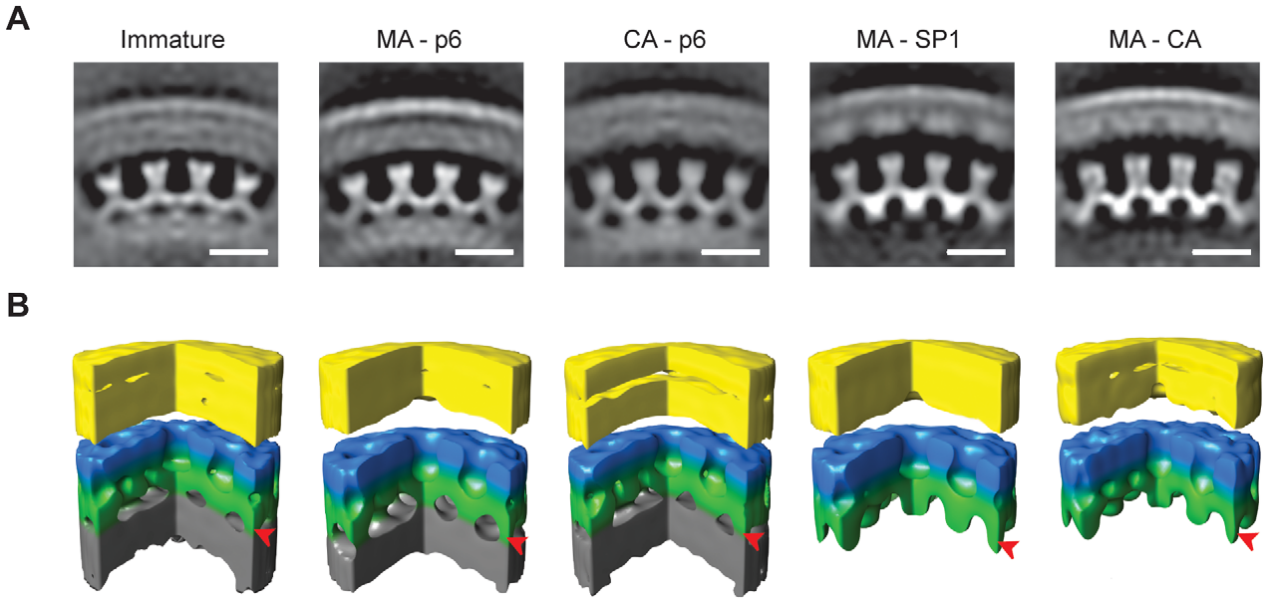
The local structure of the Gag layer. A) Radial sections from the subtomogram average reconstructions coming from each variant. Density is white. The scale bar is 10 nm. B) Surface rendering of the subtomogram average reconstructions. A sector of 90° has been cut out to better show the organization of the lattice. The colours on the surfaces are for illustrative purposes. The membrane and the MA layer, which cannot be delineated at this resolution, are yellow. N-terminal CA domain is blue. C-terminal CA domain and SP1 spacer are green. NC-RNA layer is grey. In MA-SP1 and MA-CA the grey NC-RNA layer is missing because condensation has already taken place, and rod-like protrusions are seen descending from the C-terminal CA domain towards the centre of the virus particle. Arrowheads mark the radial position of the rod-like densities.

The structure of the Gag lattice in the PR inhibited virus (Figure 4) was consistent with that previously published for immature HIV using these methods [10]. The lattice had a six-fold symmetric structure with an inter-hexagon distance of 8 nm measured at the C-terminal CA domain. The outermost density, previously assigned to the N-terminal domain of CA, formed hexameric rings around large holes. The C-terminal domain of CA sat at a lower radius at the 2-fold symmetrical position between the hexameric rings. These rings were linked at the 6-fold positions, beneath the holes in the N-terminal CA layer, where they joined a rod-like density, previously proposed to represent SP1 descending into the NC-RNA layer, which was not hexagonally ordered. At this resolution, the structure of MA–p6 was indistinguishable from the PR-inhibited wt virus.

The structure of the CA–p6 lattice showed that the outer hexameric rings corresponding to N-CA, the 2-fold densities corresponding to C-CA and the rod-like densities were all unchanged. The inter-hexagon distance remained 8 nm. These observations imply that the CA, SP1 and NC-RNA layers in the CA-p6 variant adopt a structure that is similar to that in the immature virus.

The N-CA and C-CA features of the MA-SP1 structure were similar to the PR-inhibited virus. The inter-hexagon distance remained 8 nm. The rod-like densities that descend from the CA layer towards the NC-RNA could also still be seen in this structure. Below the rod-like densities, the reconstruction of the lattice of the MA-SP1 variant lacked the inner disordered layer that in the immature virus has been assigned to the NC-RNA. The structure of the lattice in MA-CA was identical to the one in MA-SP1, despite the partial cleavage of the SP1 region.

### A link between partially cleaved Gag and the NC-RNA

All preparations contained some partially broken virus particles. Condensed NC-RNA, and sections of Gag lattice, which were not part of intact virus particles, could be visualised in the tomograms. We noticed that sections of MA-SP1 lattice from broken particles were commonly associated with condensed NC-RNA (data not shown), suggesting the possibility of an interaction between the condensed NC-RNA and the MA-SP1 lattice.

**Figure 5:**
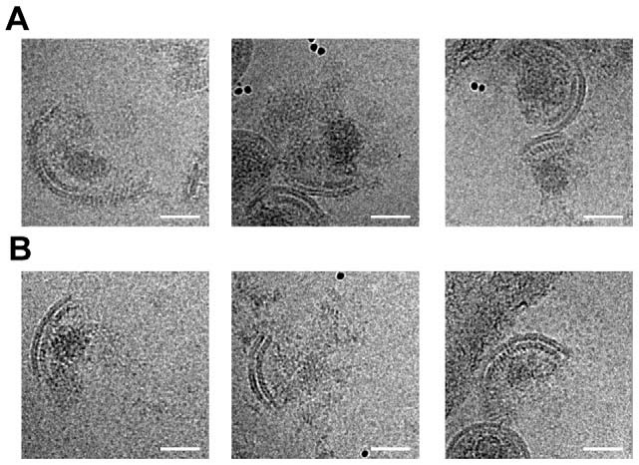
Analysis of disrupted virus particles. Micrographs of MA – SP1 (A) and MA – CA (B) virus particles after disruption by vortexing in the presence of gold beads show patches of viral membrane with underlying CA lattice. The condensed NC – RNA is visible as an irregular, globular density similar to that seen within intact virus particles (compare with figure 2B). Rather than diffusing away from the broken particles, the NC-RNA appears to remain associated with the CA lattice. The scale bar is 50 nm.

To further explore the possibility of a direct link between the condensed NC-RNA and the remaining Gag lattice, we gently disrupted MA-SP1 and MA-CA virus particles by vortexing for 15 s in the presence of 10 nm gold beads. Cryo-electron micrographs of these preparations showed large numbers of virus particles with disrupted membranes. All of the broken particles had condensed densities associated with the remaining MA-SP1 lattice (Figure 5). In some cases the presence of neighbouring particles or the orientation made interpretation difficult, but in most of the cases, the size and shape of the density suggested that it represented the condensed NC-RNA.

## Discussion

Here we have studied HIV-1 maturation by using cryo-electron tomography of a panel of viruses with mutations in proteolytic cleavage sites to describe the structures of potential maturation intermediates at macromolecular resolution. Analysis of these viruses reveals how individual cleavage events influence the structure and disassembly of the immature lattice as well as the formation of the mature lattice.

MA-p6 viruses lacking functional cleavage sites in Gag, but exhibiting wt PR activity and regular processing of Pol, present a lattice structure that is indistinguishable from immature HIV-1 produced in the presence of a PR inhibitor. The completeness of the Gag shell and the size of the viruses is also the same as for protease inhibited wt viruses. This suggests that (i) processing of the Pol domain does not detectably influence the lattice structure if Gag remains uncleaved, (ii) the alteration of cleavage sites did not have a significant effect on the structure and assembly of the Gag layer. Further analysis of variants with mutated cleavage sites revealed that processing at both sides of CA-SP1 is needed for dissolution of the immature lattice, while release of SP1 is needed, in addition, for formation of the regular mature core.

### MA-SP1 forms a structure which is stable in the absence of NC

The structure of the MA-SP1 variant shows that cleavage between SP1 and NC leads to removal of the NC-RNA layer from Gag, and to condensation of the NC-RNA core. On the other hand, the structure of the MA-SP1 region of the lattice remained unchanged compared to the immature virus. NC-RNA interactions or other non-specific interaction domains downstream of SP1 have been shown to be essential for virus-like particle assembly both in vitro and in tissue culture, however [36]. The stability of the lattice in MA-SP1 indicates that these interactions concentrate and align Gag at the membrane during assembly, but are dispensable for maintaining the structure or stability of the Gag lattice once it is formed.

The same structure as is observed here for MA-CA and MA-SP1 was also seen in a subset of cellular budding sites and released particles in vivo, (see accompanying manuscript by Carlson *et al*). This structure can therefore also be formed by wt Gag if premature SP1-NC cleavage occurs and leads to loss of PR from the budding particle. Observing the same structure also in particles carrying wt Gag polyproteins provides further support for it representing a true maturation intermediate.

In the MA-CA construct, all cleavage sites downstream of CA remain in their wt form. Nevertheless, cleavage efficiency for the CA-SP1 site is reduced to between 20% and 50%, due to the failure of cleavage between MA and CA. These observations suggest that failure to cleave upstream of CA inhibits access of PR to the CA-SP1 cleavage site as has also been reported for mutation of the downstream SP1-NC cleavage site [37]. It has been shown that cleavage at the wt CA-SP1 site is also inhibited in a *trans*-dominant way if small amounts of MA-SP1 are incorporated into HIV-1 particles [26]. Our data indicate that in all of these situations there is a failure to sufficiently disassemble the immature CA lattice (see below), and that disassembly of the immature CA layer is required for efficient access of PR to the CA-SP1 cleavage site.

Both MA-CA and MA-SP1 variants showed rod-like structures descending from the CA layer towards the centre of the virus. These rod-like structures were not disrupted by cleavage between SP1 and NC, or by subsequent partial cleavage between CA and SP1. This argues that SP1-SP1 interactions together with CA-SP1 interactions are sufficient to maintain the integrity of the rod-like structures even in the presence of partial CA-SP1 cleavage. SP1 contains important assembly determinants, and this observation suggests that these direct formation of a specific structure, which does not require NC tethering for its stability.

### CA-NC forms a structure which is stable in the absence of MA

In solution, cleavage between MA and CA has been demonstrated to cause rearrangement in the N-terminal domain of CA, leading to part of the MA-CA flexible-linker folding back into the N-terminal domain of CA to form a β-hairpin structure [20]. However in the CA-p6 variant, where this cleavage has occurred, the CA lattice was identical to the one present in the immature virus, at the resolution of the reconstructions. It also had the same overall completeness. This suggests that formation of the β-hairpin structure either requires downstream processing in addition, or that its formation is insufficient to cause structural changes in the immature lattice.

Although the structure between CA and p6 in the CA-p6 variant was the same as that in the immature virus, the distance between CA and the membrane became more variable, indicating the absence of a MA-CA link. The curvature of the CA layer was more variable than in the immature virus, suggesting that interactions with the membrane contribute to the uniformity of the lattice curvature.

### Cleavage upstream and downstream of CA-SP1 is required for immature lattice disassembly: cleavage within CA-SP1 for mature lattice assembly

As discussed above, both MA-SP1 and CA-p6 show CA lattices with an immature structure. We can therefore conclude that cleavage either only upstream or only downstream of the CA-SP1 domain does not affect the organization of the immature lattice. The immature lattice is disassembled, however, when cleavage has occurred both upstream of CA and downstream of SP1 in the case of the CA-SP1 variant, which contains no characteristic, striated CA lattice. In thin section electron micrographs, an irregular electron-dense structure separated from the virion membrane was commonly observed for this virus [27]. Such irregular layers were occasionally seen in our analyses as well, but were too thin to represent an immature CA lattice. They are more comparable in thickness to the mature CA core, which shows a thinner CA layer than the immature virus due to the closer proximity of the N- and C- terminal CA domains. Inhibition of cleavage at the CA-SP1 cleavage site therefore does not appear to prevent disassembly of the immature lattice, but to affect formation of the regular mature core.

CA-SP1 and even CA-NC constructs are able to assemble in vitro into mature-like lattices [13], [21], [36], indicating that there is no absolute requirement for cleavage at the CA-SP1 junction for mature CA lattice assembly. Indeed the thin layers sometimes seen in CA-SP1 particles may represent patches of mature CA lattice, but formation of the closed mature core around the NC-RNA complex does not occur unless SP1 is removed from CA. It is currently not clear why SP1 cleavage is needed for formation of the mature core inside the virion, while being dispensable for in vitro assembly of the mature-like lattice. We speculate that this may be due to a defect in nucleating formation of the cone-shaped mature core in the virion if SP1 is not cleaved. It is interesting, in this context, that in vitro assembly of CA or C-terminally extended versions of CA generally leads to hollow tubes, and formation of a closed cone is observed only very rarely. The in vitro system may thus not completely mimic this aspect of virion maturation. Cleavage between CA and SP1 is the site of action of the maturation inhibitor bevirimat, suggesting that this drug has no effect on disassembly of the immature lattice but acts by preventing formation of the closed cone-shaped core of the mature virion.

### A link between the condensed NC-RNA and the immature CA lattice

In the immature virus the NC-RNA is organized in a disordered but compact layer below the CA lattice. When Gag is cleaved downstream of SP1, the NC region condenses. Approximately the same degree of condensation was seen in the MA-SP1, MA-CA, CA-SP1 and wt variants, suggesting that there is no role for free CA in NC-RNA condensation. The high variability in shape and volume (if related to the size of the virus) indicates that the condensed NC-RNA does not form a well-defined structure.

MA-SP1 and MA-CA exhibited a condensed NC-RNA, as well as an intact immature CA layer. Surprisingly, broken MA-SP1 and MA-CA particles exhibited associated density consistent with NC-RNA. The size and shape of this density and the absence of any other strong candidates in the purified virus preparation suggests to us that this density is indeed NC-RNA. We expect that diffusion of the NC-RNA away from the protein shell would have occurred during the vortexing procedure used to disrupt the particles, or within the time taken to transfer the sample to the grid and prepare it for electron microscopy, if no physical linkage was present. These observations therefore suggest that the NC-RNA remains attached to the CA lattice, suggesting the presence of an interaction or link between the partially cleaved lattice and the condensed NC-RNA in these variants.

If a non-covalent interaction is present, it could represent a non-specific interaction between the NC-RNA complex and the CA lattice. The interaction could also be specific: a cellular or viral protein present within the CA lattice may expose a defined binding site for a component of the NC-RNA complex. A more attractive hypothesis is that there is a direct link between NC-RNA and CA in the form of uncleaved Gag at this position. Such a link could be locally maintained if, for example, binding of the RNA packaging signal to NC protects the SP1-NC cleavage site such that it is not accessible to the viral PR. It is tempting to speculate that maintenance of such a link during maturation may allow the NC-RNA to function as a template for formation of the conical core, and/or to ensure that the NC-RNA is packaged within the core.

In summary, our data suggest that cleavages at both sides of the CA-SP1 module are required to disassemble the immature Gag lattice with removal of the NC-p6 region initiating the independent condensation of the NC-RNA core, still connected with the membrane bound lattice. Subsequent cleavage between MA and CA causes dissociation of the immature lattice, but the resulting CA-SP1 module requires final cleavage of the SP1 peptide to permit assembly of the mature cone.

## Methods

### Constructs and virus preparation

Derivatives of proviral plasmid pNL4-3 carrying mutations at specific PR cleavage sites within Gag, as well as the PR defective variant NL4-3D25A have been described previously [26], [38].

293T cells were maintained in DMEM with 10% fetal calf serum and antibiotics. Transfections with the indicated proviral derivatives were performed using the calcium phosphate method. Culture media were harvested at 42 h post transfection cleared by low speed centrifugation (5 min, 1500 g) followed by filtration through 0.45 µM nitrocellulose filters. Particles were purified by centrifugation through a 20% (w/w) sucrose cushion and subsequent centrifugation on an Iodixanol gradient as described [39]. Purified virus was inactivated with 1% paraformaldehyde for 1 h on ice. Successful inactivation was confirmed by infection of C8166 cells and scoring for syncytia formation up to 10 days after inoculation. At least 3 independent particle preparations were analyzed for each variant shown with no variability in the structures with the exception of the CA-SP1.

### Sample preparation and data acquisition

Purified viruses were mixed with 10 nm colloidal gold particles, deposited on C-flat holey Carbon grids, and vitrified by plunge-freezing in liquid ethane. Tilt series were collected on an FEI Tecnai F30 “Polara” transmission electron microscope with Gatan GIF 2002 post column energy filter and 2 k×2 k Multiscan CCD camera. Data collection was performed at 300 kV using the SerialEM Software. Tilt series were collected between 60° and −60° with 3° angular increment; the total electron dose applied to the tomograms was approximately 90 e/Å^2^. Tomograms were acquired at defocuses between 2.6 and 6.0 µm, with a magnification of 34000X resulting in a pixel size at the specimen level of 4.0 Å.

### Image processing

Tomograms were reconstructed using IMOD [40]. Subtomogram averaging was carried out as described in [10] and below using MATLAB (Mathworks). Lattice map representations were generated using Amira (Visage Imaging), together with the EM Package [41]. A hexagon is placed at the final aligned position of each tomogram, and coloured according to the cross-correlation value between the sub-tomogram and the average. Tomograms aligned to an inappropriate radial position were excluded based on the radii distribution of the set of 20 nearest subtomograms surrounding each subtomogram. If the radius of the selected subtomogram was in the first or in the fourth quartile of the distribution the subtomogram was excluded. Only hexagons with a cross-correlation over a defined threshold are displayed. The threshold was appropriately set such that hexagons were not displayed where density corresponding to Gag was absent in the tomogram. Density maps were displayed using Amira, surface rendering was done using UCSF Chimera [42].

### Subtomogram averaging

For subtomogram averaging, tomograms collected at defocuses between 2.6 and 2.9 µm were used. Sub-volumes of (38.3 nm)^3^ were extracted from tomograms along the surface of a sphere centred in the centre of the virus and with a radius equal to the mean radius at CA level. The sub-volumes were iteratively aligned. The initial reference used for the alignment was the average of the subtomograms in the extraction position. All variants subjected to sub-tomogram averaging show 6-fold symmetry, as evidenced using radius-angle-frequency plots (see Text S1 and Figure S2). 6-fold symmetry was therefore applied to the average after all iterations. The threshold for the subtomograms to be averaged was set to the mean cross-correlation value between all subtomograms and the reference. The final reconstructions had a resolution according to the Fourier shell correlation with a 0.5 criterion of approximately 26.5 Å in the CA region (Figure S3A) and are filtered to this resolution. The resolution varies with radius, with the highest resolution in the CA region (Figure S3B).

## Supporting Information

Text S1 Supplementary methods.
(0.05 MB PDF)

Figure S1 The morphology of virus variants. Central sections of tomographic reconstructions of the HIV-1 variants analyzed acquired at different defocuses (df), and coming from 3 different preparations, to illustrate consistency of virus morphology between preparations. A gaussian filter was applied the tomograms (8 kernel, 0.4 sigma). The scale bar is 50 nm.
(5.19 MB TIF)

Figure S2 Radius-angle-frequency plots. A) 3D view of the radius-angle-frequency plot calculated from the immature HIV data, illustrating the relationship between the three axes. Three perpendicular sections are shown intersecting at the point: 53 nm radius, 60° angle and 7 nm frequency. The colour bar represents the value of autocorrelation and is common to all the panels in the figure. The presence of a peak at a particular point in radius, frequency and angle indicates that at that radius in the virus, the 2D power spectrum of the protein layer has peaks at that frequency which are arranged rotationally symmetrically repeating at that angle (see supplementary methods). B–F) Data from 5 variants. The top panel is a section at the radius where the C-CA domain is found showing two peaks at 60° and 120°, with 7 nm frequency, as expected from a hexagonal unit cell with 8 nm spacing (see supplementary methods). The peaks at 0 and 180° are seen in all 2D power spectra since power spectra have intrinsic 2-fold symmetry. The middle panel is a section at 60° angle that shows that the 7 nm peak is extended across the CA region. The third panel is the radial density profile of the virus in the regions containing Gag. Starting outside the virus (high radius) the first two peaks, typically between 55 and 65 nm, represent the two leaflets of the bilayer and the associated MA, the next two peaks, typically between 45 and 55 nm, represent CA, and the peak below 45 nm, where present, represents the NC - RNA.
(2.11 MB TIF)

Figure S3 Fourier shell correlation. A) Fourier shell correlation plots for all the variants. The resolution was determined as the frequency at which the FSC curve drops below 0.5 correlation which is highlighted with the dashed line. B) Plot showing the variation in resolution according to radius. At each radius the resolution was determined by Fourier shell correlation at 0.5, with a mask centred at that radius (see supplementary methods). The positions of NC -RNA, CA, MA and membrane are indicated.
(0.89 MB TIF)
