# Supplementary material for: Structural Analysis of HIV-1 Maturation Using Cryo-Electron Tomography
Source: PLoS Pathog. 2010 Nov 24;6(11):e1001215. doi: 10.1371/journal.ppat.1001215 (PMC2999899; doi:10.1371/journal.ppat.1001215)
Supplement: Text S1 — Supplementary methods. (0.05 MB PDF) [file ppat.1001215.s001.pdf]

Text S1:

Radius-angle-frequency plots

The same subtomograms used in the subtomogram averaging were iteratively aligned allowing only translations in Z (no rotations or translations in other directions were allowed, no symmetry was applied). The starting reference used was the average of the subtomograms in the extraction position. A mask that included the CA layer and the membrane was applied during alignment. Only the subtomograms that contained Gag (to exclude those from regions of the surface where no Gag layer was present) were used for the analysis; this was done by considering only the subtomograms selected for the lattice maps.

Aligned subtomograms underwent a radial orthographic projection. The focus of the projection was determined as the geometric centre of the virus from which the subtomogram was extracted.

The resulting volumes corresponded to flattened subtomograms. 2D power spectra were calculated at each radius. The rotation-autocorrelation function with a rotation range between 0° and 180° was calculated for each power spectrum. For each subtomogram this generates a 3D plot with radius on one axis, and the other two axes representing those of the rotational autocorrelation function of the power spectrum, namely angle and frequency. This analysis is a 3D implementation and combination of the analyses presented in figures 3c and 5 of [1]. The 3D plots for all aligned, selected subtomograms were averaged to generate the final plot.

Radial density profiles

The flattened subtomograms used for the radius-angle-frequency plots were averaged, and the mean density value at each radius was plotted against radius.

Fourier shell correlation (FSC)

The dataset was split two halves, and two averages where generated. To avoid the possibility of an artificial resolution measurement, where multiple subtomograms had shifted and rotated during the alignment to bring their overlapping regions together, all but one were discarded. Since the resolution has variability across the lattice the averages were masked with a soft mask that included only the CA region, and the FSC was calculated.

In order to define the variation of the resolution according to radius, the same mask used for the calculation of the resolution in CA was shifted by increments of 1 pixel, and the resolution at which the FSC curve dropped below 0.5 for each radius was plotted.

References

1. Briggs JA, Johnson MC, Simon MN, Fuller SD, Vogt VM (2006) Cryo-electron microscopy reveals conserved and divergent features of gag packing in immature particles of Rous sarcoma virus and human immunodeficiency virus. J Mol Biol 355: 157-168.
