# Supplementary figures and images for: Structural Analysis of HIV-1 Maturation Using Cryo-Electron Tomography
Source: PLoS Pathog. 2010 Nov 24;6(11):e1001215. doi: 10.1371/journal.ppat.1001215 (PMC2999899; doi:10.1371/journal.ppat.1001215)

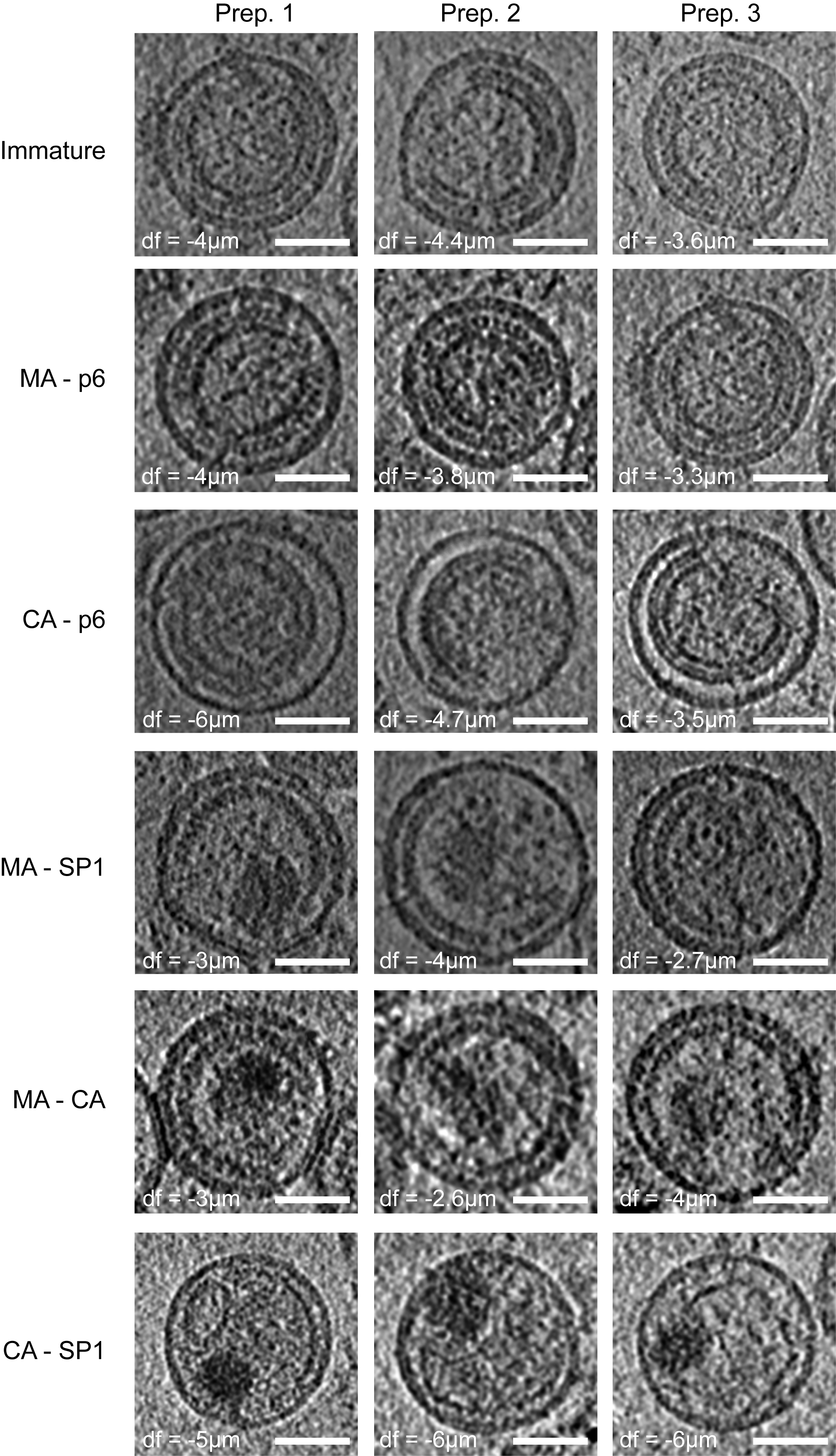

Supplement: Figure S1 — The morphology of virus variants. Central sections of tomographic reconstructions of the HIV-1 variants analyzed acquired at different defocuses (df), and coming from 3 different preparations, to illustrate consistency of virus morphology between preparations. A gaussian filter was applied the tomograms (8 kernel, 0.4 sigma). The scale bar is 50 nm. (5.19 MB TIF) [file ppat.1001215.s002.tif]

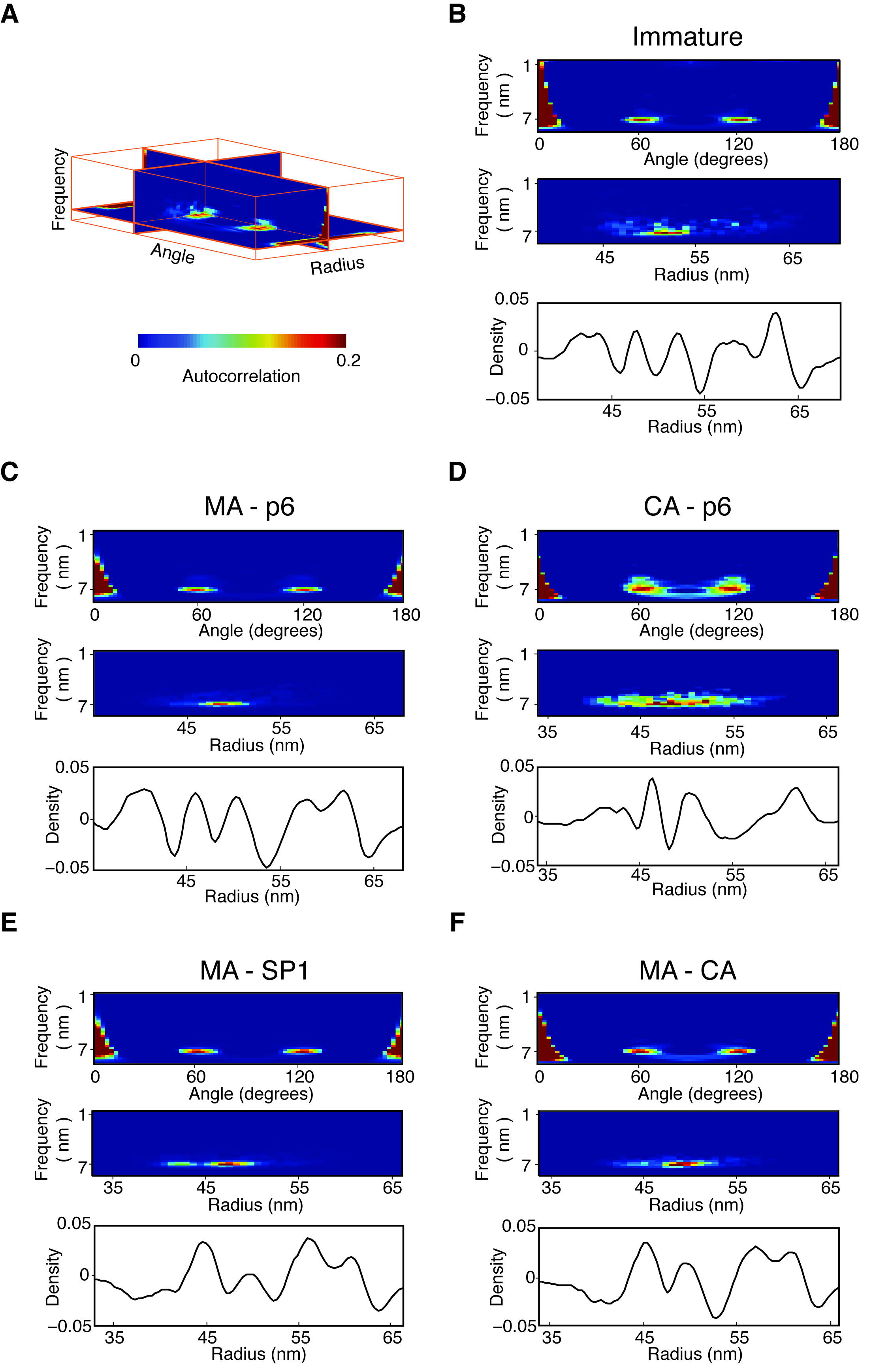

Supplement: Figure S2 — Radius-angle-frequency plots. A) 3D view of the radius-angle-frequency plot calculated from the immature HIV data, illustrating the relationship between the three axes. Three perpendicular sections are shown intersecting at the point: 53 nm radius, 60° angle and 7 nm frequency. The colour bar represents the value of autocorrelation and is common to all the panels in the figure. The presence of a peak at a particular point in radius, frequency and angle indicates that at that radius in the virus, the 2D power spectrum of the protein layer has peaks at that frequency which are arranged rotationally symmetrically repeating at that angle (see supplementary methods). B–F) Data from 5 variants. The top panel is a section at the radius where the C-CA domain is found showing two peaks at 60° and 120°, with 7 nm frequency, as expected from a hexagonal unit cell with 8 nm spacing (see supplementary methods). The peaks at 0 and 180° are seen in all 2D power spectra since power spectra have intrinsic 2-fold symmetry. The middle panel is a section at 60° angle that shows that the 7 nm peak is extended across the CA region. The third panel is the radial density profile of the virus in the regions containing Gag. Starting outside the virus (high radius) the first two peaks, typically between 55 and 65 nm, represent the two leaflets of the bilayer and the associated MA, the next two peaks, typically between 45 and 55 nm, represent CA, and the peak below 45 nm, where present, represents the NC - RNA. (2.11 MB TIF) [file ppat.1001215.s003.tif]

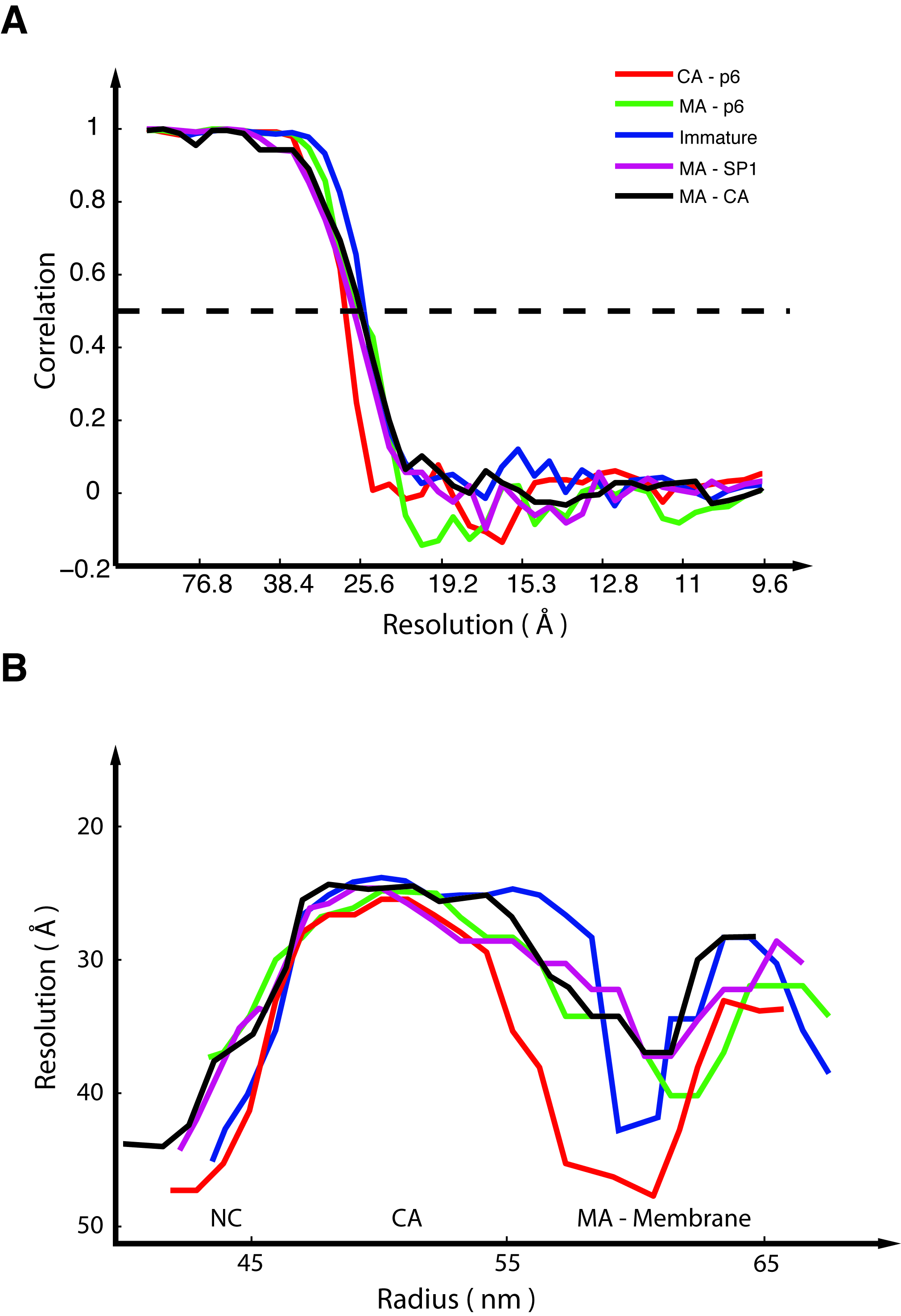

Supplement: Figure S3 — Fourier shell correlation. A) Fourier shell correlation plots for all the variants. The resolution was determined as the frequency at which the FSC curve drops below 0.5 correlation which is highlighted with the dashed line. B) Plot showing the variation in resolution according to radius. At each radius the resolution was determined by Fourier shell correlation at 0.5, with a mask centred at that radius (see supplementary methods). The positions of NC -RNA, CA, MA and membrane are indicated. (0.89 MB TIF) [file ppat.1001215.s004.tif]
